# Health insurance provider and endovascular treatment availability are associated with different hemodialysis vascular access profiles: A Brazilian national survey

**DOI:** 10.3389/fneph.2022.985449

**Published:** 2022-12-07

**Authors:** Ricardo Portiolli Franco, Domingos Candiota Chula, Thyago Proença de Moraes, Rodrigo Peixoto Campos

**Affiliations:** ^1^ Interventional Nephrology Center, Fundação Pró-Renal, Curitiba, Brazil; ^2^ Department of Nephrology, Pontifícia Universidade Católica do Paraná (PUCPR), Curitiba, Brazil; ^3^ Department of Nephrology, Santa Casa de Misericórdia de Curitiba, Curitiba, Brazil; ^4^ Department of Internal Medicine, Faculdade de Medicina da Universidade Federal de Alagoas, Maceió, Brazil

**Keywords:** dialysis, arteriovenous fistula, angioplasty, endovascular procedures, catheter, vascular access

## Abstract

In Brazil, most hemodialysis (HD) patients are treated by the country’s public health system. However, accessibility to healthcare is different for public and private patients. This study aimed to identify the profile of vascular access in a Brazilian HD sample. Additionally, it aimed to examine the influence of public and private health insurance, accessibility to endovascular treatments, and timely arteriovenous access creation on the prevalence of tunneled catheters (TCs), non-tunneled catheters (NTCs), and arteriovenous (AV) access. We conducted a cross-sectional electronic survey across 834 centers. Centers were inquired about the number of patients with public and private health insurance, the profiles of vascular access, time for AV access creation, accessibility to TC insertion and endovascular treatments, and the availability of peritoneal dialysis and kidney transplantation. Logistic regression and multilevel logistic regression were performed to evaluate possible interactions between the independent variables. A total of 7,973 patients across 47 HD centers were included in the survey. Public health patients accounted for 77% of the study sample. The overall vascular access profiles of the public and private insurance groups were significantly different (*p* < 0.001). For patients with public health insurance, the prevalence of any catheter was 25%, while that for private patients was 31.8% (*p* < 0.001). The prevalence of TCs was more common in private patients (15.3% vs. 23.1%, *p* < 0.001). AV accesses were more common in public health patients (75% vs. 68.2%, *p* < 0.001), as were fistulas (72.4% vs. 63.1%, *p* < 0.001). AV grafts were more prevalent among patients with private insurance (2.6 vs. 5.1%, *p* < 0.001). The availability of endovascular treatments increased the chance of having a TC by 2.3-fold (OR = 2.33, 95% CI = 1.30–4.18); however, it did not reduce the chance of having any catheter. A high chance of having a catheter was found when the time to AV access creation exceeded 60 days. The differences between public and private patients may be explained by underpayments and the decreased accessibility to care infrastructure in the public system, especially for endovascular treatments. In this sample, public health patients had a decreased risk of having a TC over an NTC. Differences in care accessibility and insurance type might influence the type of vascular access.

## Introduction

According to the Brazilian Dialysis Census, Brazil had around 148,000 dialysis patients in 2021, with a 56% increase in the last decade (1). As many as 94% of these patients are treated with hemodialysis (HD), of which 82% come under the public health system called SUS (*Sistema Único de Saúde*). Hemodialysis is dependent on a functional vascular access, and for most patients, an arteriovenous fistula (AVF) or arteriovenous graft (AVG) is often preferred over catheters. Catheters, especially non-tunneled catheters (NTCs), cause more infections and hospitalizations and have shorter survival rates than arteriovenous (AV) accesses, leading to higher costs and morbidity (2–6). The Kidney Disease Outcomes Quality Initiative (KDOQI) guidelines suggest that the NTC should not be used beyond 2 weeks (7). Chronic HD patients mostly use tunneled catheters (TCs) as the preferred type of catheter since it has a long subcutaneous tunnel with a Dacron cuff to anchor, which reduces the risk of infection.

Despite the established role of TCs in HD, high rates of NTC use are common in Brazilian HD centers, and catheter prevalence has been rising steadily. In Brazil, catheter prevalence increased from 15.4% to 23.9% from 2013 to 2021, mainly due to an increase in TCs. Nevertheless, 8.6% of all catheters in 2021 were NTCs, and AVF prevalence decreased from 81.3% to 73.9% in the same period (1).

Vascular access is a complex part of HD and demands considerable infrastructural advancement and personnel training to follow international guidelines. Brazil has a universal public health system that covers the majority of HD patients. However, in many regions, practicing nephrologists and vascular surgeons face difficulties in switching from NTCs to TCs, attaining AV access creation, and carrying out endovascular procedures in the public health system. These difficulties may have an influence on the use of the different types of vascular access.

As the official Brazilian Dialysis Census only collects data on the type of vascular access, we decided to perform a survey among Brazilian HD centers aiming to identify vascular access profiles and their associations with the type of healthcare insurance and the characteristics of treatment availability.

## Materials and methods

### Study design

We conducted a cross-sectional survey to determine the characteristics of vascular access in Brazilian HD centers in February 2022. An electronic survey comprising 21 questions uploaded on Google Forms was sent *via* e-mail to the HD centers. At the time, 834 HD centers were registered with the Brazilian Society of Nephrology. The person in charge of vascular access control at the HD center was required to respond to the survey.

The questionnaire sought information on the size of the center, the distribution of patients with public and private insurance, profiles of vascular access in patients with public and private health insurance, time for AV access (AVF or AVG) creation, accessibility to TC insertion and endovascular treatments (including angioplasties and thrombectomies), specialties involved in vascular access creation, and the availability of peritoneal dialysis (PD) and kidney transplantation. The main questions sent to the clinics are summarized in **Table 1**. The study was approved by the local Research Ethics Committee (authorization no. 57815722.3.0000.5013).

**Table 1:** Survey questions about vascular access profile and care accessibility.

Number of patients (public and private)
Number of patients with AVF, AVG, TC, and NTC for public and private patients
Time from HD initiation to AVG/AVG creation for public and private patients
Routine use of TC until AVF or AVG of public and private patients (yes/no)
Availability of endovascular procedures in cases of vascular access dysfunction in public and private patients (yes/no)
Specialists involved in TC insertion, AVF/AVG creation, and endovascular procedures
Availability of PD and renal transplantation (yes/no)

AVF, arteriovenous fistula; AVG, arteriovenous graft; TC, tunneled catheter; NTC, non-tunneled catheter; PD, peritoneal dialysis

### Objectives

The main objective of this study was to evaluate the profiles of vascular access in a Brazilian HD clinic sample and the factors associated with the presence of NTCs, TCs, and AV accesses. Furthermore, we aimed to investigate whether accessibility to endovascular procedures, the type of healthcare insurance, and the time taken to create AV access had an impact on the use of HD catheters.

### Statistical analysis

Continuous variables were described as the mean ± standard deviation or median (first and third quartiles) depending on their distribution. Categorical variables were described as absolute numbers and percentages. Student’s *t*-test was applied to normally distributed variables and the Mann–Whitney *U* test for the non-normally distributed variables. Pearson’s chi-square test was used for categorical variables. To evaluate the potential factors associated with the presence of a catheter for current vascular access, we first used logistic regression. We evaluated all potential confounders obtained in the survey, and those with a *p*-value lower than 0.20 were selected to be part of a full multivariate model. Thereafter, in a stepwise process, we began excluding non-significant predictors, beginning with those with a higher alpha value, until reaching the final level. We compared the model with the best fit using the maximum likelihood test. Furthermore, we considered the hierarchical structure of the data where the patient was at the first level, the dialysis clinic at the second level, and the region of the country at the third level. Thereafter, a mixed-model analysis for the same binary outcome was performed. Finally, to investigate the potential interaction between the prevalence of catheters, we stratified the analysis by region, availability of PD and transplantation, as well as endovascular treatments in the center, and the time taken to create AV access. The equation used and the model fitted are available in a **supplementary file**. All analyses were performed using STATA 16. The alpha level for significance was set at 0.05.

## Results

A total of 47 HD centers responded to the survey, resulting in a sample of 7,973 patients. **Tables 2** and **3** show the characteristics of the patients. Most HD centers (66%) were located in the southern and southeastern regions. The distribution of our sample was significantly different from that of the official Census (*p* < 0.001). **Table 4** shows the distribution of the HD clinics in the official national society data and our survey. Patients with public and private health insurance were found in 74.5% of the centers. The prevalence of TCs was found in 17%, NTCs in 9.6%, and AV access in 73.4% of patients. Endovascular procedures were available for 52.9% of the patients. The time taken to create AV access was up to 30 days in 39.48% of patients; it was between 30 and 60 days in 28.02% of patients. No difference between patients with public and private insurance was observed for AV access creation time higher than 60 days.

**Table 2:** Clinical and demographic characteristics of the study population.

Variable	Total population (7,973 patients)
Centers, *n*	47
Patients per center, *n* ^a^	154 (113–210)
Non-tunneled catheter, *n* (%)	764 (9.6)
Tunneled catheter, *n* (%)	1,357 (17.0)
AVF, *n* (%)	5,606 (70.3)
AVG, *n* (%)	246 (3.1)
Health insurance care, *n* (%)	
Public Private Mixed	7 (14.9) 5 (10.6) 35 (74.5)
Endovascular available, *n* (%)	4,220 (52.9)
PD available, *n* (%)	30 (63.8)
Transplant available, *n* (%)	28 (59.6)
Time (days) to AV access creation, *n* (%)	
<30 30–60 61–90 91–120 >120	3,148 (39.48) 2,234 (28.02) 1,026 (12.87) 1,135 (14.24) 430 (5.39)

AVF, arteriovenous fistula; AVG, arteriovenous graft; PD, peritoneal dialysis; AV, arteriovenous

a Median (first and third quartiles).

**Table 3:** Clinical and demographic characteristics of the patients stratified by health insurance provider.

Variable	Public	Private	*p*-value
Patients, *n* (%)	6,140 (77)	1,833 (23)	
Center size, *n* (%)			<0.001
<150 150–250 >250	1,433 (23.5) 3,164 (51.5) 1,533 (25.0)	561 (30.6) 740 (40.4) 532 (29.0)	
Region, *n* (%)			<0.001
South Southeast Midwest Northeast North	2,198 (35.7) 2,220 (36.1) 771 (12.6) 447 (7.3) 504 (8.2)	509 (27.8) 589 (32.1) 310 (16.9) 60 (3.3) 365 (21)	
Vascular access, *n* (%)			<0.001
Non-tunneled catheter Tunneled catheter Arteriovenous fistula Arteriovenous graft	604 (9.8) 934 (15.2) 4,449 (72.4) 153 (2.6)	160 (8.7) 423 (23.1) 1157 (63.1) 93 (5.1)	
Endovascular available, *n* (%)	2,819 (45.9)	1,401 (76.4)	<0.001
Time (days) to AV access creation, *n* (%)			<0.001
<30 30–60 61–90 91–120 >120	2,549 (41.5) 1,584 (25.8) 844 (13.7) 740 (12.1) 423 (6.9)	599 (32.7) 650 (35.5) 182 (9.9) 395 (21.5) 7 (0.4)	

AV, arteriovenous

**Table 4:** Number of active dialysis clinics and patients of the official Census and the study survey.

	Active centers in 2021	Responding centers		Patients	
		Survey*	Census 2021	Survey*	Census 2021
*N*	849	47	252	7,973	44,037
Region, *n* (%)					
South Southeast Midwest Northeast North	153 (18.0) 403 (47.5) 78 (9.2) 163 (19.2) 52 (6.1)	18 (38.3) 13 (27.7) 8 (17.0) 3 (6.4) 5 (10.6)	59 (23.4) 113 (44.8) 24 (9.5) 42 (16,6) 14 (5.5)	2,707 (34) 2,809 (35.2) 1,081 (13.6) 507 (6.4) 889 (11.2)	8,251 (18.7) 21,059 (47.8) 3,729 (8.5) 9,082 (20.6) 1,916 (4.4)

*Mann-Whitney U test, p<0.02.

The overall vascular access profiles were significantly different between the public and private insurance groups (*p* < 0.001). For patients with public health insurance, the prevalence of any type of catheter was 25%, while that for private patients was 31.8% (*p* < 0.001). For NTCs, the difference was not statistically significant between the two groups (9.8% vs. 8.7%); however, TCs were more frequent in patients with private insurance (15.3% vs. 23.1%, p<0.001). In public health patients, AV access (AVF or AVG) was significantly more frequent (75% vs. 68.2%, *p* < 0.001), as was AVF (72.4% vs. 63.1%, *p* < 0.001). However, in patients with private insurance, AVG was more prevalent (2.6% vs. 5.1%, *p* < 0.001).

The prevalence of patients under public health insurance was higher in HD centers with 150–250 patients (*p* < 0.001). Furthermore, the number of patients under private health insurance was higher in the North and Midwest regions (*p* < 0.001). **Table 5** shows the survey and overall private insurance coverage rates by region and the findings on the accessibility to endovascular treatments for both health systems (8). Endovascular treatment was more commonly available to patients with private health insurance (45.6% vs. 76.4%, *p* < 0.001).

**Table 5:** Rates of private insurance coverage and availability of endovascular procedures by region.

Region	Private insurance rate on survey (%)	Official private insurance coverage (%)	Availability of endovascular procedures on public health system (%)	Availability of endovascular procedures on private health system (%)
South Southeast Midwest Northeast North	18.8 20.9 28.6 11.8 42	24.9 35.1 21.4 12.2 10.6	57.1 44.4 16.6 33.3 75	80 100 75 100 0

In 49% of the centers, a nephrologist was involved in the insertion of TCs. However, that did not significantly affect the vascular access profiles of the participants. Nephrologists were involved in endovascular treatments in only three centers, all in the same city.

**Figure 1:**
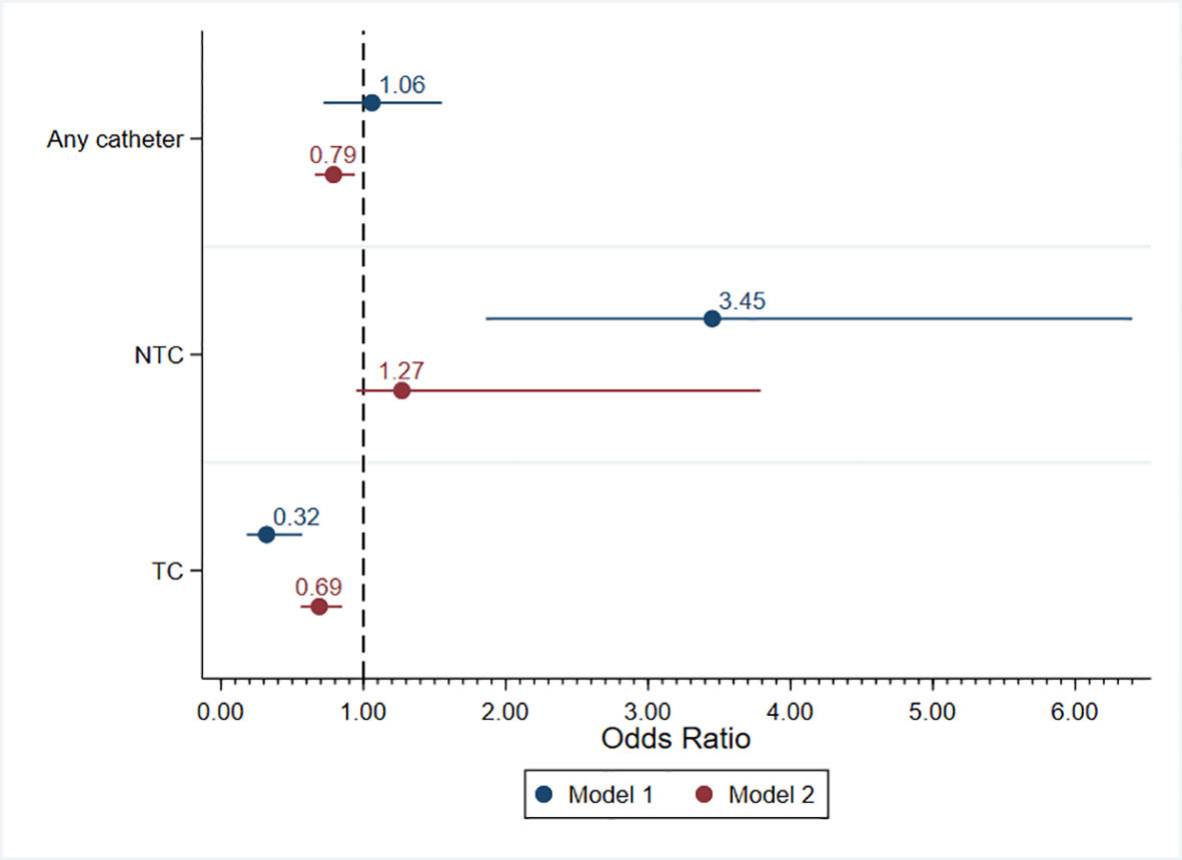
Multilevel logistic regression models for the risk of having a catheter. *NTC*, non-tunneled catheter; *TC*, tunneled catheter.

**Tables 6** and **7** list the results of the two multilevel logistic regression models for the risk of having a catheter. The two models were necessary given the collinearity between the two center characteristics, namely, the availability of endovascular treatments and the time taken to create AV access. In the first model, which included the type of health insurance, HD center region, center size, and the availability of endovascular treatment, the chance of having an NTC for patients with public health insurance was 3.4-fold greater (OR = 3.45, 95% CI = 1.86–6.40). Compared to the southern region, patients from the Midwest had a 51% lower chance of having a catheter (OR = 0.49, 95% CI = 0.29–0.83). The availability of endovascular treatments increased the chance of having a TC by 2.3-fold (OR = 2.33, 95% CI = 1.30–4.18), but did not reduce the chance of having any catheter. The second model, which included the type of health insurance, HD center region, center size, and the time taken to create AV access, demonstrated a decreased chance of having a catheter by 21% for patients with public health insurance (OR = 0.79, 95% CI = 0.66–0.94), possibly due to the reduced risk of having a TC (OR = 0.69, 95% CI = 0.56–0.85). Patients treated at centers located in the Midwest had a 43% lower chance of having a catheter (OR = 0.43, 95% CI = 0.25–0.74). A higher chance of having a catheter, especially an NTC, was found when the time to create AV access exceeded 60 days (**Table 7**). **Figure 1** summarizes the odds of having a catheter as vascular access found on both models.

**Table 6:** Model 1 for adjusted multilevel logistic regression for the risk of having a hemodialysis (HD) catheter.

	Type of catheter		
	Any catheter	NTC	TC
	OR (95% CI)	OR (95% CI)	OR (95% CI)
Health insurance			
Public	1.06 (0.72–1.55)	***3.45 (1.86–6.40)***	***0.32 (0.18–0.57)***
Region			
South Southeast Midwest Northeast North	Reference 0.87 (0.54–1.40) ***0.49 (0.29–0.83)*** 0.86 (0.40–1.83) 1.18 (0.65–2.16)	Reference 0.60 (0.26–1.40) *0.92 (0.37–2.25)* 1.05 (0.29–3.82) 1.61 (0.57–4.59)	Reference 0.71 (0.33–1.55) ***0.33 (0.14–0.79)*** 0.59 (0.17–2.00) 0.71 (0.26–1.91)
Center size (*n* patients)			
<150 150–250 >250	Reference 0.79 (0.53–1.18) 1.39 (0.76–2.55)	Reference 1.18 (0.59–2.36) 0.55 (0.18–1.61)	Reference 0.62 (0.33–1.20) 2.08 (0.78–5.57)
Endovascular treatment			
Yes	0.76 (0.52–1.13)	***0.29 (0.15–0.54)***	***2.33 (1.30–4.18)***

OR, odds ratio; NTC, non-tunneled catheter; TC, tunneled catheter. Bold values are statistically significant odds ratios.

**Table 7:** Model 2 for adjusted multilevel logistic regression for the risk of having a hemodialysis (HD) catheter.

	Type of catheter		
	Any catheter	NTC	TC
	OR (95% CI)	OR (95% CI)	OR (95% CI)
Health insurance			
Public	***0.79 (0.66–0.94)***	1.27 (0.95–3.79)	***0.69 (0.56–0.85)***
Region			
South Southeast Midwest Northeast North	Reference 0.94 (0.60–1.45) ***0.43 (0.25–0.74)*** 0.76 (0.38–1.53) 0.80 (0.43–1.48)	Reference 0.69 (0.26–1.83) ***0.71 (1.05–7.20)*** 0.63 (0.14–2.81) 0.54 (0.14–2.05)	Reference 0.65 (0.28–1.47) ***0.34 (0.13–0.86)*** 0.77 (0.21–2.80) 0.93 (0.30–2.86)
Center size (*n* patients)			
<150 150–250 >250	Reference 0.80 (0.55–1.17) 1.34 (0.75–2.37)	Reference 1.39 (0.61–3.18) 0.50 (0.14–1.79)	Reference 0.54 (0.27–1.09) 2.28 (0.80–6.52)
Time to AV access creation			
<30 30–60 61–90 91–120 >120	Reference 1.32 (0.94–1.87) ***1.72 (1.10–2.69)*** ***2.28 (1.58–3.29)*** 1.85 (0.98–3.48)	Reference 1.90 (0.96–3.79) ***2.75 (1.05–7.20)*** ***7.04 (3.69–13.4)*** ***6.05 (1.79–20.5)***	Reference 0.73 (0.42–1.25) 0.92 (0.49–1.76) 0.61 (0.35–1.09) 0.54 (0.19–1.49)

OR, odds ratio; NTC, non-tunneled catheter; TC, tunneled catheter; AV, arteriovenous. Bold values are statistically significant odds ratios.

## Discussion

This study provides an overview of the factors that may influence the prevalence of the different types of vascular access in a Brazilian HD sample randomly distributed across the country, which considered mainly public and private health insurance coverage and care accessibility. We observed an elevated prevalence of catheters, and the results suggest a possible association between public health insurance and a reduced chance of using a TC. Furthermore, the availability of endovascular treatments was associated with an increased risk of having a TC; moreover, the greater the delay to create AV access, the higher the chance of having an NTC and the lesser the chance of achieving AV access.

Although this randomly distributed sample may not be fully representative of the Brazilian dialysis population, the official Brazilian dialysis Census was filled out by only 252 of the 849 (29.7%) active dialysis clinics in 2021. This comprises 44,037 patients and our sample of 7,973 patients. The low rate of respondents in the official Census raises concern about the need for stronger data on vascular accesses. In addition, the only official data about vascular access include the prevalent modality, as the Census did not split the data into regions or provider type and did not evaluate care accessibility. Nevertheless, the distribution of the vascular access types found in this survey was very similar to that of the Brazilian dialysis Census: AVF = 70.3% vs. 73%, AVG = 3.1% vs. 2.3%, TC = 17% vs. 17.1%, and NTC = 9.6% vs. 7.6% (1). As shown in **Table 4**, the distribution of clinics in this study differed from that of the official Census. Furthermore, the north region had a much higher rate of patients with private insurance in our survey than the overall official data (42% vs. 10.6%).

A recent cross-sectional study in a northeast state of Brazil, which included 2,513 HD patients (18 HD centers), found an AVF prevalence of 79.6%, AVG of 0.9%, NTC of 15.0%, and TC of 4.5%. Nevertheless, in the study sample, catheters constituted 89% of the initial vascular access cases (9). In 2003, Linardi et al. reported vascular access prevalence from 2,559 patients (23 HD centers) in Brazil, as follows: AVF = 91.2%, AVG = 3.2%, NTC = 5.4%, and TC = 1.2% (10). According to the Brazilian Society of Nephrology annual surveys and other Brazilian reports, the prevalence of catheters, mainly TCs, is growing while the prevalence of AVF is decreasing in country. The prevalence of vascular access in our study was similar to those reported in previous studies.

A Brazilian cross-sectional study comprising 2,276 patients reported a prevalence of 69% for NTCs at HD initiation. The risk factors for NTC use include pre-dialysis care for less than 1 year and lack of private insurance (11). A retrospective analysis of 5,081 Brazilian patients, 58.1% of whom were covered by the public health insurance system, found that, for 69.6% of patients, dialysis was initiated with a catheter (56% NTC and 13.6% TC) (12). Compared to patients with private insurance, those with public health insurance had more AVF (31.9 vs. 27%, *p* < 0.0001), less TCs (6.2 vs. 23.8%, *p* < 0.0001), and more NTCs (61.5% vs. 48.5%, *p* < 0.0001). Patients with private insurance were older and were more frequently referred earlier to nephrologists. In this study, having a catheter as the first access and an unplanned HD start were associated with a higher risk of death within 5 years. Other studies also found high rates of NTC use in Brazilian public health patients (13). According to our findings, public health insurance may possibly affect the type of prevalent vascular access, increasing the chance of having an NTC and reducing the chance of TC use. Patients with public health insurance may have lesser access to an early nephrological consultation and, consequently, might face a delay in AV access creation, thereby providing a greater chance of NTC exposure. The lack of availability of TCs can lead to vascular access site exhaustion due to the need for frequent NTC removal or exchange in cases of dysfunction and infection. The use of TCs tends to help the patient with a longer survival rate with fewer infections and dysfunctions, preserving available vascular access sites, whereas the use of NTCs increases the risk of bloodstream infections and morbidity in HD patients (2–6, 14).

Variation in vascular access is undoubtedly one of the most important determinants of patient outcomes, but the vascular access profiles differ greatly between countries. Japan and Russia have more than 90% AVF use. However, in 2013, the prevalence of AVF varied between 49% and 92% across 20 countries [Japan, China, Europe, Australia/New Zealand, Canada, the USA, and the Gulf Cooperation Council (GCC)]. The UK and the USA have reduced catheter use by <20%. Contrastingly, in Canada and Belgium, catheter use has increased to 40% (15).

In Brazil, more than 80% of patients on HD are covered by public health insurance (1). This was reflected by our study findings, wherein 77% of HD patients were under public health insurance. Most HD centers provide care for patients with either public or private insurance (74.5%). Thus, for patients with public insurance, accessibility to TC and endovascular treatments and faster AV access creation should be facilitated. Nevertheless, these findings might raise the concern that patients with public health insurance may have an inferior chance of having a TC, which may be explained by the decreased availability of endovascular suites and specialists. In Brazil, the reimbursement paid by the public health insurance system is insufficient for the TC insertion procedure and the catheter itself. In our opinion, this is probably the main reason for the elevated prevalence of NTC catheter use in Brazilian HD centers. Moreover, inferior access to adequate infrastructure, such as ultrasound machines and fluoroscopy suites, and the low training opportunities for nephrologists and surgeons exacerbate the problem. Despite these barriers, TC prevalence has increased over the years, which may be explained by the increasing interventional nephrology and HD vascular access training in the country (16, 17). In our survey, nephrologists were involved in TC catheter procedures in almost half the centers; however, their participation in endovascular procedures was very low (3 out of 47 centers), probably because of the limited access to endovascular suites and training. Nephrologist participation as an interventionist was not associated with the prevalence of TC use. In a study that surveyed 239 Brazilian nephrologists in 2006, only 23% of the respondents judged themselves trained enough to insert a TC, and only 47% were trained to place an NTC (18).

Care accessibility may also explain the differences in the prevalence of AVF and AVG between the patients with public and private insurance in our sample. In this study, patients from the public health insurance system had a significantly higher prevalence of AVF and lower prevalence of AVG. In Brazil, AVF can be created in an outpatient setting for public health patients and is reimbursed by the public health insurance system. However, in many centers, the use of AVG is limited due to the need for hospitalization, which may take longer in the public health system. Furthermore, public health system reimbursement for graft materials is insufficient. Moreover, despite the higher maturation rates of AVG, grafts develop stenosis and thrombosis earlier and more often than AVF (19). The lack of accessibility to endovascular treatments in these cases invariably leads to access loss.. In practice, the use of an AVG with no possibility of treatment of venous anastomosis stenosis may have an extremely low chance of long access survival. As other studies have pointed out, patients with private insurance tend to be older in Brazil, and as surgeons have easier access to AVG for this group, they may use it as suggested by the KDOQI guidelines (7, 20, 21). In private practice, if an AVG fails, most patients may have access to endovascular treatments.

A Chinese study that evaluated the impact of two different public health systems—one prominently covering urban areas and the other covering rural areas—found that patients covered by the rural health system had more catheter and less AVF use and had a high overall mortality rate. The use of catheters was an independent predictor of all-cause mortality (22). Similar to China, Brazil, too, has several disparities between urban and rural areas.

Although international guidelines suggest TCs for patients with catheter dependence for more than 2 weeks, along with image guidance for TC insertion and endovascular treatments for dysfunctional accesses (7), these resources are not often available in low-income regions covered mainly by the public health system in Brazil. In our sample, only 45.9% of the patients with public health insurance had access to endovascular procedures. Our findings may suggest that a lack of availability of endovascular treatments increases the risk of having NTCs. On multilevel logistic regression, the availability of endovascular therapy was associated with a 71% decrease in the chance of having an NTC. In the same model, public health patients demonstrated a 3.4-fold greater chance of having an NTC. Even though in Brazil TCs are often inserted without fluoroscopy due to the lack of infrastructure, specific situations, such as difficult cases, central stenosis, and patients with multiple previous accesses, need fluoroscopy in order to be dealt with and to have a TC properly placed. Furthermore, centers that do not offer endovascular treatments have fewer treatment options for AV access stenosis and thrombosis.

A South African report found that 56% of patients had catheters, despite only 12% of patients not being submitted to AV access creation attempt. Almost a third of the patients in this study waited more than 12 months prior to the first AVF attempt, and a 26% prevalence of central venous stenosis or occlusions was reported (23). The authors commented on the lack of infrastructure for HD vascular access, especially surgical and endovascular suites, and how the lack of endovascular treatments undermines the creation of vascular accesses, as up to 46% would need an intervention to mature. The impossibility of treating primary failures may lead to the creation of a new AV access, reducing the available creation sites and leading to longer catheter exposure and potential complications (24–28). Brazil has no regulations for outpatient endovascular procedures; therefore, patients with dysfunctional access need to be hospitalized for treatment. As most vascular access angioplasties are considered non-urgent procedures, reports of months of delays—from the diagnosis of stenosis to treatment—are common in the public health system. Such long periods of wait are not practical for HD patients, as many develop thrombosis and require a catheter until a new AV access is created. Most vascular access thrombosis is due to stenosis, which can be treated with angioplasty without disrupting the dialysis schedule of the patient (16, 17, 29–31). In the USA, the outpatient treatment of dysfunctional vascular access in specialized centers is a consolidated reality, with published results (32–35).

The creation of regional reference centers to centralize vascular access procedures could also increase the use of AV access. A report including 74 Veterans Administration Medical centers and 1,114 patients found the odds of having AVF as first access to be three times higher at the center, with >30 initial vascular access procedures per year compared to those with less than 10. Additionally, they found a strong clustering effect at the surgeon level, suggesting that different personal practice patterns can affect vascular access use (36).

Our study had other limitations beyond selection bias. We did not evaluate the social, economic, or clinical characteristics of the patients, which can affect the choice and outcomes of vascular access. We have included the region as an independent variable in the attempt to mitigate this limitation. The annual Brazilian dialysis Census collects limited data on vascular access, focusing on the prevalence of TC, NTC, and AV accesses, and is filled out by roughly 30% of the country’s dialysis clinics. Furthermore, the data available from the Census did not split the vascular access profiles by region. Therefore, the profiles shown by the Brazilian Society of Nephrology, as the one in this report, may be biased, as centers with better care are more inclined to submit their data. Only 5.6% of the HD clinics responded to our survey, and the geographic distribution and private insurance coverage rates differed from the official Census. Therefore, it is important to collect more comprehensive and detailed data on vascular access to guide care policies. Despite these important limitations, our survey included a considerable number of patients and was the first to evaluate health insurance providers, availability of endovascular treatments, and time to AV access creation as predictors of a prevalent catheter.

Our findings in this randomly distributed sample may suggest a gap in care between public and private practices, as public health patients had a decreased risk of having a TC. Although a difference is expected in the use of expensive and advanced materials, such as drug-coated balloons and stents, it is concerning that patients with public health insurance, who represent a majority in our country, may be exposed to risks of complications due to a higher exposure to NTCs. Larger studies on care accessibility and stronger official data are needed to guide future vascular access policies in Brazil. Spreading vascular access care through training, adequate reimbursement, and facilitation of accessibility to infrastructure should be considered.

## Data availability statement

The raw data supporting the conclusions of this article will be made available under request to the corresponding author.

## Ethics statement

The studies involving human participants were reviewed and approved by the Universidade Federal de Alagoas [authorization no. (CAAE) 57815722.3.0000.5013]. The patients/participants provided written informed consent to participate in this study.

## Author contributions

TM provided statistical advice and performed the analysis. All authors contributed to the article and approved the submitted version.
