# Supplementary material for: Health insurance provider and endovascular treatment availability are associated with different hemodialysis vascular access profiles: A Brazilian national survey
Source: Front Nephrol. 2022 Dec 7;2:985449. doi: 10.3389/fneph.2022.985449 (PMC10479601; doi:10.3389/fneph.2022.985449)

Supplemental file 1

The equation used for the hierarchical model follows a special case of binomial data (Bernouili), as described in the original STATA document, page 22 ([*https://www.stata.com/manuals/memelogit.pdf*](https://www.stata.com/manuals/memelogit.pdf)).

Code used:

“*melogit Tunelizado SUS1_Conv0 i.Regiao i.Center_Size i.Time_AVF_short || Clinic_ID : ,or* “

The output of the tunneled catheter outcome (Table 4 3^rd^ column) is pasted here for a better comprehension:


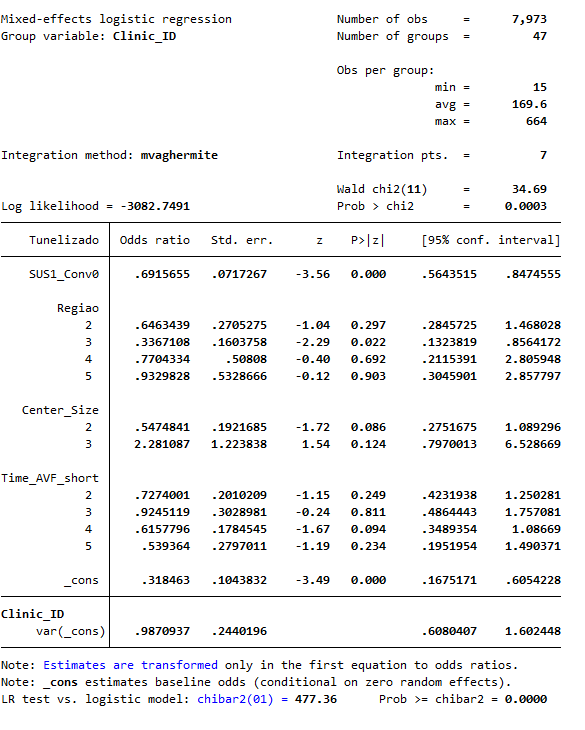

Supplement: Supplementary file 1 [file Table_1.docx]
